# Chondrogenic differentiation of bone marrow-derived mesenchymal stem cells following transfection with Indian hedgehog and sonic hedgehog using a rotary cell culture system

**DOI:** 10.1186/s11658-019-0144-2

**Published:** 2019-02-26

**Authors:** Liyang Chen, Gejun Liu, Wenjun Li, Xing Wu

**Affiliations:** Department of Orthopedics, Shanghai Tenth People’s Hospital, School of Medicine, Tongji University, Shanghai, 200072 People’s Republic of China

**Keywords:** Indian hedgehog, Sonic hedgehog, RCCS, Chondrogenic differentiation, Chondrocyte hypertrophy

## Abstract

**Background:**

Indian hedgehog (IHH) and Sonic hedgehog (SHH) are important regulators of chondrogenesis. However, activation of IHH and SHH also promotes chondrocyte hypertrophy and ossification during chondrogenesis. The aims of this study were to investigate the effect of microgravity on IHH- and SHH-induced chondrogenic differentiation and to elucidate the role of microgravity in this process.

**Methods:**

Adenovirus plasmids encoding the rabbit IHH gene and SHH genes were constructed in vitro and transfected into rabbit bone marrow-derived mesenchymal stem cells (BMSCs). A rotary cell culture system (RCCS), in which a dynamic three-dimensional culture system combines the mechanical environment with a three-dimensional culture surface, was used for cell culture and differentiation. During the induction of differentiation, expression levels of cartilage-related and cartilage hypertrophy-related genes and proteins were detected by quantitative real-time polymerase chain reaction (qRT-PCR) and western blotting, respectively. Toluidine blue and collagen II immunohistochemical staining and annexin V-Cy3 staining were used to indicate investigate cartilage matrix synthesis and hypertrophic hypertrophy, respectively, on day 21 after induction of differentiation.

**Results:**

In this study, IHH and SHH were shown to be equipotent inducers of chondrogenesis in rabbit BMSCs, as evidenced by strong staining for proteoglycans and collagen II, and increased expression of mRNAs and proteins associated with chondrogenesis in an RCCS environment. More importantly, chondrogenic hypertrophy and aging were effectively inhibited in the RCCS environment. In addition, levels of cartilage-related markers in the IHH and SHH transfection groups were initially increased and later decreased in the traditional two-dimensional environment, while cartilage hypertrophy-related factors revealed higher mRNA expression levels during induction.

**Conclusions:**

In summary, microgravity significantly promoted chondrogenic differentiation of BMSCs induced by IHH and SHH and attenuated chondrogenic hypertrophy and aging during chondrogenesis. Furthermore, exogenous IHH and SHH had the same effect on chondrogenic differentiation of BMSCs in the RCCS environment. This study provides further evidence of chondrogenic induction of BMSCs in vitro via IHH and SHH gene delivery.

**Electronic supplementary material:**

The online version of this article (10.1186/s11658-019-0144-2) contains supplementary material, which is available to authorized users.

## Background

Excessive physical activity at all age levels has been associated with increased incidence of articular cartilage injuries, causing significant pain and functional disability. The self-healing capability of articular cartilage is limited due to its limited vascular supply and inability to undergo chondrocyte proliferation [1]. Joint articular cartilage degeneration plays a fundamental role in the pathogenesis of osteoarthritis (OA), which is characterized by joint pain, stiffness, and limited mobility [2]. Bone marrow-derived mesenchymal stem cells (BMSCs) have been widely used in tissue regeneration due to their potential to differentiate into multiple cell lineages including osteocytes, adipocytes, and chondrocytes [3, 4]. However, BMSC-based cartilage tissue regeneration biochemically and biomechanically adequate for clinical use has not been successful and is hindered by chondrogenic induction and hypertrophy. Lin et al. observed severe OA in mice genetically modified to express elevated hedgehog signaling, and inhibition of hedgehog signaling in murine systems and human cartilage explants alleviates OA-derived cartilage damage [5]. A recent study by Wu et al. revealed that both SHH and IHH mRNA are expressed in postnatal growth plate (GP) chondrocytes [6]. These findings suggested that hedgehog signaling may promote differentiation of chondrogenic precursor cells [7, 8].

Hedgehog proteins constitute a conserved family of macromolecules that provide crucial embryonic patterning signals in many organisms. Higher vertebrates express at least three hedgehog proteins, including Sonic hedgehog (SHH), Indian hedgehog (IHH), and desert hedgehog [6]. One important role of SHH involves the regulation of anterior–posterior patterning in avian and mammalian limb development, and at the early bud stage of limb development, SHH is the only expressed member of the hedgehog family. Murine SHH knockout models display extremely foreshortened limbs, complete absence of vertebrae, and loss of distal structures, which suggest that SHH plays important roles in skeletogenesis of developing limbs [9]. IHH, a homolog of SHH, appears in the middle of the condensing cartilage elements at later stages of embryonic development during the formation of skeletal elements [10]. However, activation of IHH and SHH also promotes chondrocyte hypertrophy and ossification during chondrogenesis [11, 12]. Chondrocyte hypertrophy is closely related to inflammation in OA. Thus, it can be seen that inhibition of pathological chondrocyte hypertrophy induced by IHH and SHH is important.

Appropriate mechanical stimulation can induce the differentiation of BMSCs into chondrocytes and allow synthesis of the extracellular matrix (ECM) [13]. The RCCS is a new three-dimensional microgravity culture system that allows culture materials to establish a suspension track similar to a homogeneous fluid, and the gravity, buoyancy, and shear force can achieve a balance, which constitutes a microgravity environment conducive to cell aggregation [14, 15]. The correct hydrostatic pressure and shear stress aid in maintaining the phenotype and function of cartilage cells.

In the present study, rabbit BMSCs were modified with adenoviral vectors encoding IHH or SHH, cultured, and induced in an RCCS and a traditional 2D environment. The goals of this study were to investigate the effect of microgravity on IHH- and SHH-induced chondrogenic differentiation and to elucidate the role of microgravity in this process.

## Materials and methods

### Construction of recombinant SHH and IHH adenovirus plasmids

PDC316-mCMV-ZsGreen1 was used as a shuttle plasmid, as a backbone vector, and for enzymatic digestion. Primers were designed and synthesized to amplify a fragment of the target gene, rabbit SHH (gene ID: 100352774). The constructed adenovirus overexpression plasmid and backbone plasmid were co-transfected into HEK293 cells for viral packaging. The viral solution was concentrated to 10^11^ plaque-forming units (pfu)/ml by ultrafiltration concentration. The adenovirus plasmids containing IHH or enhanced green fluorescence protein (GFP) were obtained from Liu et al. [14], and the titer was 1 × 10^11^ pfu/ml.

### Cell culture and grouping

BMSCs were obtained from the femur and tibia of ten female New Zealand white rabbits (4 weeks old), purchased from Shanghai Jambo Biological Technology Co., Ltd. (Shanghai, China). All experimental procedures were approved by the Care of Experimental Animals Committee of the Tenth People’s Hospital of Tongji University, China. Cultures were started in 2 ml of low glucose Dulbecco’s Modified Eagle Medium (L-DMEM) (Gibco, Grand Island, NY, USA) with 10% fetal bovine serum (Gibco, Grand Island, NY, USA) in a 5% CO_2_:95% air atmosphere. BMSCs at passage 2 were evaluated for homogeneity via CD73, CD90, CD105, CD44, CD34, CD19 and CD45 detection using flow cytometry. BMSCs were verified on the basis of their ability to differentiate into osteocytes, chondrocytes and adipocytes (Additional file 1: Figure S1). BMSCs were used for viral transfection and induction of chondrogenesis at passage 2 [14]. The culture medium for chondrogenesis was composed of H-DMEM (Hyclone, Pittsburgh, USA), 1% insulin-transferrin-selenium solution (Gibco, Grand Island, USA), 50 μg/ml ascorbate, 10^− 7^ M dexamethasone, 100 μg/ml sodium pyruvate, and 40 μg/ml L-proline (all from Sigma-Aldrich, St. Louis, USA), and 1% penicillin–streptomycin (Gibco, Grand Island, USA).

The experiment was divided into two groups: the RCCS group and the 2D culture group. Each group was subsequently divided into four smaller groups: IHH transfection, SHH transfection, GFP transfection, and non-transfection.

### Transfection of adenovirus plasmids into rabbit BMSCs and induction of chondrogenic differentiation

Transfection methods were performed as described by Liu et al. [14]. Rabbit BMSCs were transfected with IHH, SHH, or GFP adenovirus plasmid at 50 pfu/cell. After 4 h of transfection, the medium was replaced with fresh BMSC cell culture medium. After 24 h of transfection, cells from each group were collected using 0.25% trypsin-EDTA solution and counted. For the 2D culture group, 3 × 10^5^ cells/well were plated into six-well plates. For all groups, the culture medium was replaced with chondrocyte differentiation medium after 24 h, and subsequently replaced every 2 days for 21 days to perform chondrogenic differentiation.

For the RCCS group, sterilized Cytodex 3 microcarriers (GE Healthcare Life Sciences, Little Chalfont, UK) were used to provide a suitable cell culture surface. Cytodex 3 is a nonporous, surface-type microcarrier that consists of a surface layer of denatured collagen covalently bound to a matrix of cross-linked dextran. Cytodex 3 microcarriers were prepared as previously described [16]. Microcarriers with dry weight of 50 mg were placed into 15-ml clean glass bottles, with 10 ml of PBS (Ca^2+^ and Mg^2+^ free, pH 7.4), and incubated at room temperature for at least 3 h to expand the microcarriers. After hydration, the PBS was removed and replaced with fresh PBS, and the microcarriers were sterilized by autoclaving at 121 °C for 20 min. Prior to use, the supernatant liquid was removed and the microcarriers were cleaned with culture liquid. They were then stored in a refrigerator at 4 °C.

The cell suspension and Cytodex 3 were fully mixed to achieve a final cell density and microcarrier concentration of 4 × 10^5^ cells/ml and 5 mg/ml, respectively. RCCS cell culture containers (10 ml) were used to culture the mixture. Following installation on the rotating base, the containers were placed in a 5% CO_2_ incubator. The rotation speed of the RCCS container was adjusted to approximately 10–12 rpm to allow full contact between the cells and the microcarriers. After 24 h, the rotation speed of the container was increased to 12–14 rpm so that the culture material in the container no longer came into contact with the container wall and remained in free-fall during rotation. For all groups, the culture medium was replaced with chondrocyte differentiation medium after 24 h and the culture medium was replaced every 2 days for 21 days. When samples required removal from the container for evaluation, the power was first turned off, after which the container was placed in a biosafety cabinet for manipulation.

### Measurement of SHH and IHH expression

The mRNA expression levels of SHH and IHH were measured by quantitative real-time polymerase chain reaction (qRT-PCR). Three samples from each group were selected at random. The protein levels of SHH and IHH were measured by enzyme-linked immunosorbent assay (ELISA). Five samples from each group were randomly selected to determine the IHH and SHH concentrations in the cell culture medium using ELISA (rabbit IHH and SHH protein ELISA, Laibio, China) as previously described [17].

### RNA isolation and reverse transcription analysis

RNA was prepared from all groups on days 7, 14, and 21. Total RNA was extracted from cells with TRIzol reagent (Invitrogen, Karlsruhe, Germany). The concentration and integrity of RNA were estimated by evaluation of the A260/280 absorbance ratio. Reverse transcription was carried out with 1 μg of total RNA from each sample using a PrimeScript RT-PCR kit (TaKaRa, China). RT-PCR reactions were performed using KAPA SYBR FAST qPCR Master Mix (2×) with High ROX (50×) (Kapa Biosystems, Wilmington, MA, USA) with the following conditions: 95 °C for 3 min followed by 40 cycles of 95 °C for 3 s and 60 °C for 30 s. Expression of Sox9, collagen II, aggrecan (ACAN), collagen X, Runx2, and annexin V in each sample was analyzed according to the real-time PCR instructions. Relative gene expression was calculated using the 2^−ΔΔCt^ method expressed as the mean of triplicate samples. Beta-2-microglobulin was used as the internal control. All primer sequences used in this study are described in Table 1.

**Table 1:** Primer sequences for quantitative real-time polymerase chain reaction

Gene	Forward primer	Reverse primer
IHH	CCAACTACAATCCCGACATCATCT	CTCGTCCCAGCCTTCAGTCA
SHH	CTGACCGTGACCGTAGCAAGT	TGGATGTGGGCTTTGGACTCA
Sox9	CTGACCGTGACCGTAGCAAGT	TGGATGTGGGCTTTGGACTCA
ANCN	ATGGCTTCCACCAGTGCG	CGGATGCCGTAGGTTCTCA
collagen II	GCTCCCAGAACATCACCTACCA	ATTCCTGCTCAGGCCCTCC
collagen X	CCCTTCTGCTGCTAGTGTC	GTCTTGGTGTTGGGTTGT
Runx2	CCTTCCACTCTCAGTAAGAAGA	TAAGTAAAGGTGGCTGGATAGT
annexin V	GCAGAACTAACAGCCATAA	AGAACCACCAACATCCTC
Beta-2-microglobulin	AACGTGGAACAGTCAGACC	AGTAATCTCGATCCCATTTC

### Western blotting

Expression of the hedgehog signaling molecules Patched (Ptc), Smoothened (Smo), and Gli1 was determined by western blotting. Expression of the cartilage-related proteins collagen II and ACAN was determined by western blotting following induction on days 10 and 21. Three samples from each group were randomly selected to extract cellular proteins. Glyceraldehyde 3-phosphate dehydrogenase (GAPDH) was used as the internal control protein. The primary antibodies for Ptc (1:500, orb19265) and Smo (1:1600, orb19363) were purchased from Biorbyt (Cambridge, UK). Gli1 (1:1000, ARP32368_T100) was purchased from Aviva Systems Biology (San Diego, CA, USA). Primary antibodies against Runx2 (1:500, ab23981) and collagen X (1:500, ab58632) were purchased from Abcam (Cambridge, UK). Primary antibodies against collagen II (1:200, NB600–844), ACAN (1:100, NB600–504), and GAPDH (1:2000, NB300–328) were purchased from Novus Biologicals (Littleton, CO, USA). HRP-conjugated anti-rabbit IgG, HRP-conjugated anti-mouse IgG, and HRP-conjugated anti-goat IgG antibodies were used as secondary antibodies at a 1:2000 dilution and were obtained from Thermo Fisher Scientific (Waltham, MA, USA).

### Histological analysis and annexin V-Cy3 staining

On day 21 after induction of differentiation, toluidine blue and collagen II immunohistochemical staining was used to investigate chondrogenesis, and annexin V-Cy3 immunofluorescence staining was used to investigate chondrocyte aging. When chondrocytes become hypertrophic or apoptotic, annexin V expression increases and, using annexin V-Cy3 staining, can be quantified as red fluorescence using an inverted fluorescence microscope [18, 19]. Toluidine blue staining was performed according to the chondrocyte toluidine blue staining reagent kit (Sigma-Aldrich, St. Louis, MO, USA), and annexin V-Cy3 staining was performed according to the annexin V-Cy3 immunofluorescence staining reagent kit (Enzo Life Sciences, Farmingdale, NY, USA). For collagen II immunohistochemical staining, endogenous peroxidase activity was blocked with 0.3% hydrogen peroxide in phosphate-buffered saline (PBS) after cells were inoculated onto slides. After blocking with goat serum (1:100), slides were incubated with collagen II antibody (1:100; Novus Biologicals) overnight at 4 °C. After washing three times with PBS, slides were incubated with the secondary antibody for 1 h at 37 °C. After a complete wash in PBS, the slides were developed in freshly prepared diaminobenzidine solution.

### ALP activity

An alkaline phosphatase assay kit (Beyotime Biotech Inc., Jiangsu, China) was used to further quantify ALP activity according to the manufacturer’s instructions following induction on days 7, 14, and 21. For quantitative ALP measurements, BMSCs were lysed in radioimmunoprecipitation lysis buffer, and the cell supernatant was collected into a 96-well plate. Following co-incubation of substrates and *p*-nitrophenol for 30 min at 37 °C, ALP activity was determined at 405 nm. Data were normalized to the concentration of total protein.

### Statistical analysis

All experiments were performed at least three times and the results were similar between repeats. All data are expressed as the mean ± standard deviation. The significance of differences between data sets was determined by analysis of variance tests followed by Student–Newman–Keuls multiple comparison and non-parametric tests using SPSS 19 statistical software (SPSS Inc., Chicago, IL, USA). *P* < 0.05 was considered significant.

## Results

### BMSC morphology and viability in cell culture

**Fig. 1:**
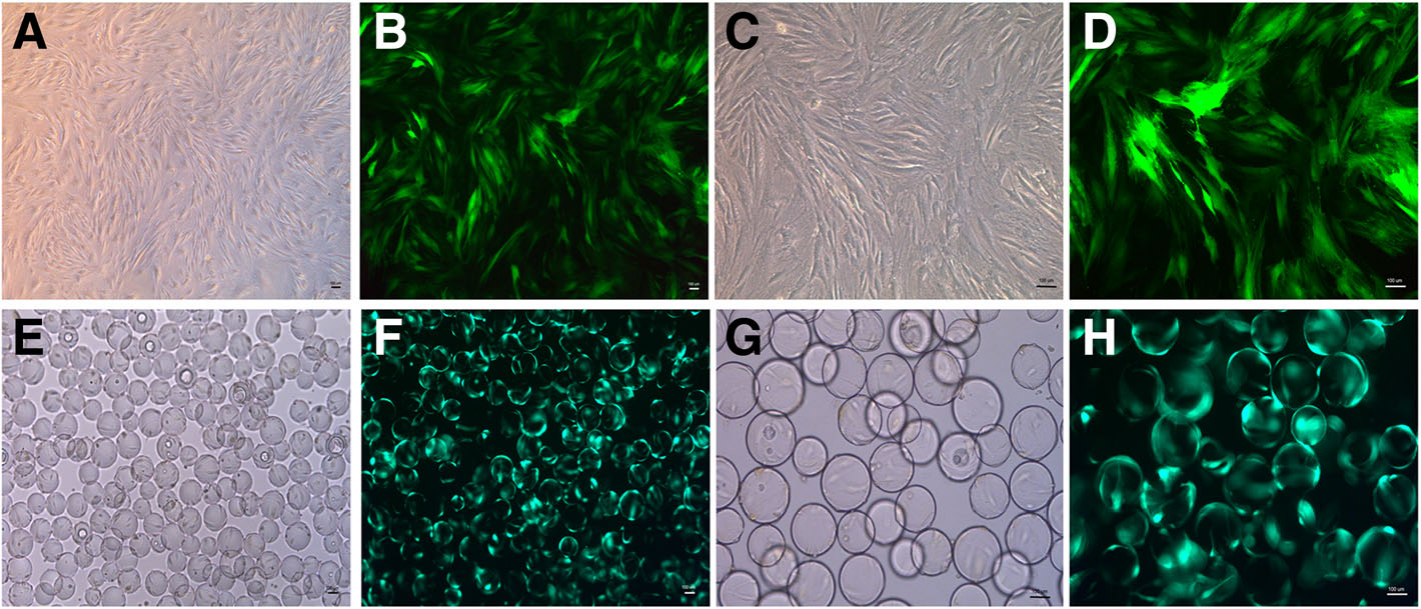
BMSC morphology. (**a** and **c**) BMSC morphology observed via inverted microscope in the 2D environment; (**b** and **d**) BMSC morphology observed via fluorescence microscopy after viral transfection; (**e** and **g**) BMSCs adhere to the surface of the Cytodex 3 microcarriers observed via an inverted microscope; (**f** and **h**) BMSCs adhere to the surface of the microcarriers via fluorescence microscopy after viral transfection. (Bar = 100 um)

In the 2D culture group, BMSCs displayed swirl-like growth at low magnification, and showed spindle-like shapes at high magnification (Fig. 1a and c). Green fluorescence observed through an inverted fluorescence microscope at 48 h after viral transfection suggested that viral plasmids were able to transfect rabbit BMSCs with high efficiency (Fig. 1b and d).

In the RCCS group, BMSCs were incubated in an RCCS bioreactor with a Cytodex 3 microcarrier surface. Cytodex 3 is a spherical cell culture microcarrier, the surface of which contains a thin layer of collagen that allows adhesion of BMSCs easily. Morphology of BMSCs following adherence to the surface of Cytodex 3 microcarriers was observed using an inverted microscope (Fig. 1e and g), and green fluorescence was observed following viral transfection (Fig. 1f and h).

### Expression of hedgehog proteins and hedgehog signaling molecules in BMSCs following viral transfection

**Fig. 2:**
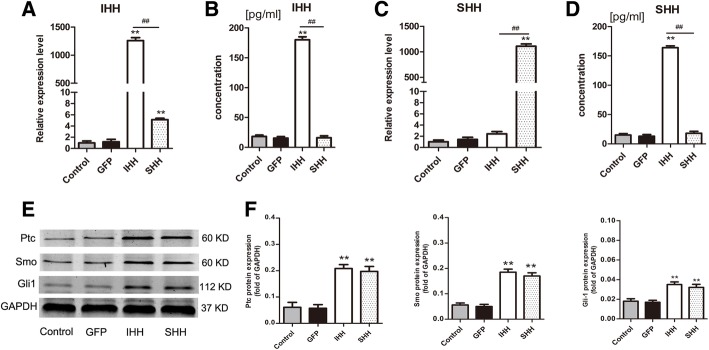
Expression levels of Hedgehog proteins and Hedgehog signaling molecules. (**a** and **c**) qRT-PCR was used to demonstrate mRNA expression levels of IHH and SHH following viral transfection; values are means ± SD (*n* = 3). The results were normalized to B2M mRNA expression. (**b** and **d**) ELISA was used to demonstrate IHH and SHH expression levels following viral transfection. Values are means ± SD (*n* = 5). (**e**) The hedgehog signaling molecules Ptc, Smo and Gli1 were detected by western blotting after viral transfection for 72 h. (**f**) The results were normalized to GAPDH protein expression. Values are means ± SD (*n* = 3). Significant differences between the control group (non-transfection cells) are indicated by *p < 0.05 or ** *p* < 0.01; differences between IHH and SHH transfection groups are indicated by ^#^ *p* < 0.05 or ^##^ *p* < 0.01

qRT-PCR and ELISA were used to evaluate the expression levels of IHH and SHH following viral transfection. The results showed that IHH and SHH were significantly upregulated in the IHH and SHH transfection groups, respectively, compared with expression levels in the GFP and non-transfection control groups (*P* < 0.01) (Fig. 2a–d).

Due to high expression levels of IHH and SHH upon transfection, we hypothesized that hedgehog signaling molecules were upregulated. The activation of Ptc, Smo, and Gli1 was assessed following viral transfection at 72 h (Fig. 2e). Expression levels of Ptc, Smo, and Gli1 in the IHH and SHH transfection groups were significantly higher than in the GFP and non-transfection groups (*P* < 0.01) (Fig. 2e and f). Levels of Ptc, Smo, and Gli1 were not significantly different between the IHH and SHH transfection groups (*P* > 0.05).

### Cartilage synthesis- and cartilage hypertrophy-related gene expression during the induction of chondrogenic differentiation

**Fig. 3:**
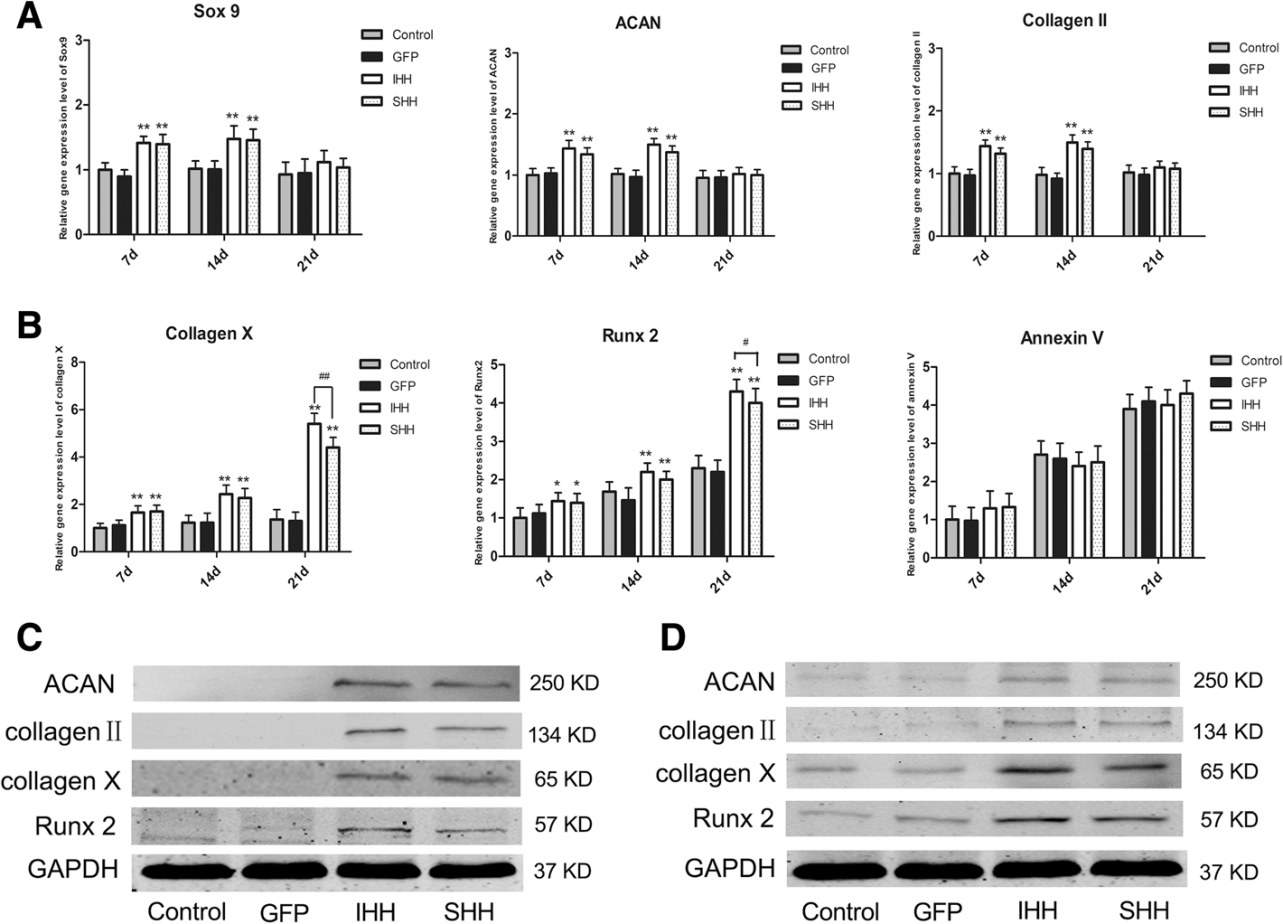
Expression levels of related genes during differentiation induction in the 2D environment. (**a**) qRT-PCR analysis of Sox9, ACAN and collagen II on days 7, 14 and 21 during induction. (**b**) qRT-PCR analysis of collagen X, Runx2 and annexin V on days 7, 14 and 21 during induction. The results were normalized to B2M mRNA expression. Values are means ± SD (n = 3). (**c**) Expression of ACAN, collagen II, collagen X and Runx2 was detected by western blotting on day 10 during induction. (**d**) Expression of ACAN, collagen II, collagen X and Runx2 was detected by western blotting on day 21 during induction. Significant differences from the control group (non-transfection cells) are indicated by *p < 0.05 or **p < 0.01; differences between IHH and SHH transfection groups are indicated by ^#^*p* < 0.05 or ^##^*p* < 0.01

We investigated the cartilage synthesis-related genes Sox9, collagen II, and ACAN, and the chondrocyte hypertrophy and aging-related genes collagen X, Runx2, and annexin V using quantitative real-time PCR. In the 2D culture group, the mRNA levels of Sox9, collagen II, and ACAN in the IHH and SHH transfection groups initially increased and later decreased. We found that the levels of Sox9, collagen II, and ACAN in the IHH and SHH transfection groups were higher than those in the non-transfection and GFP transfection groups on days 7 and 14 following the induction of differentiation (*P* < 0.01) but lower at day 21 (Fig. 3a). At the same time, the mRNA expression levels of collagen X, Runx2, and annexin V in the IHH and SHH transfection groups were significantly increased during the induction of differentiation (Fig. 3b). In addition, the levels of collagen X and Runx2 in the IHH transfection group were higher than those in the SHH transfection group on day 21 (*P* < 0.05).

**Fig. 4:**
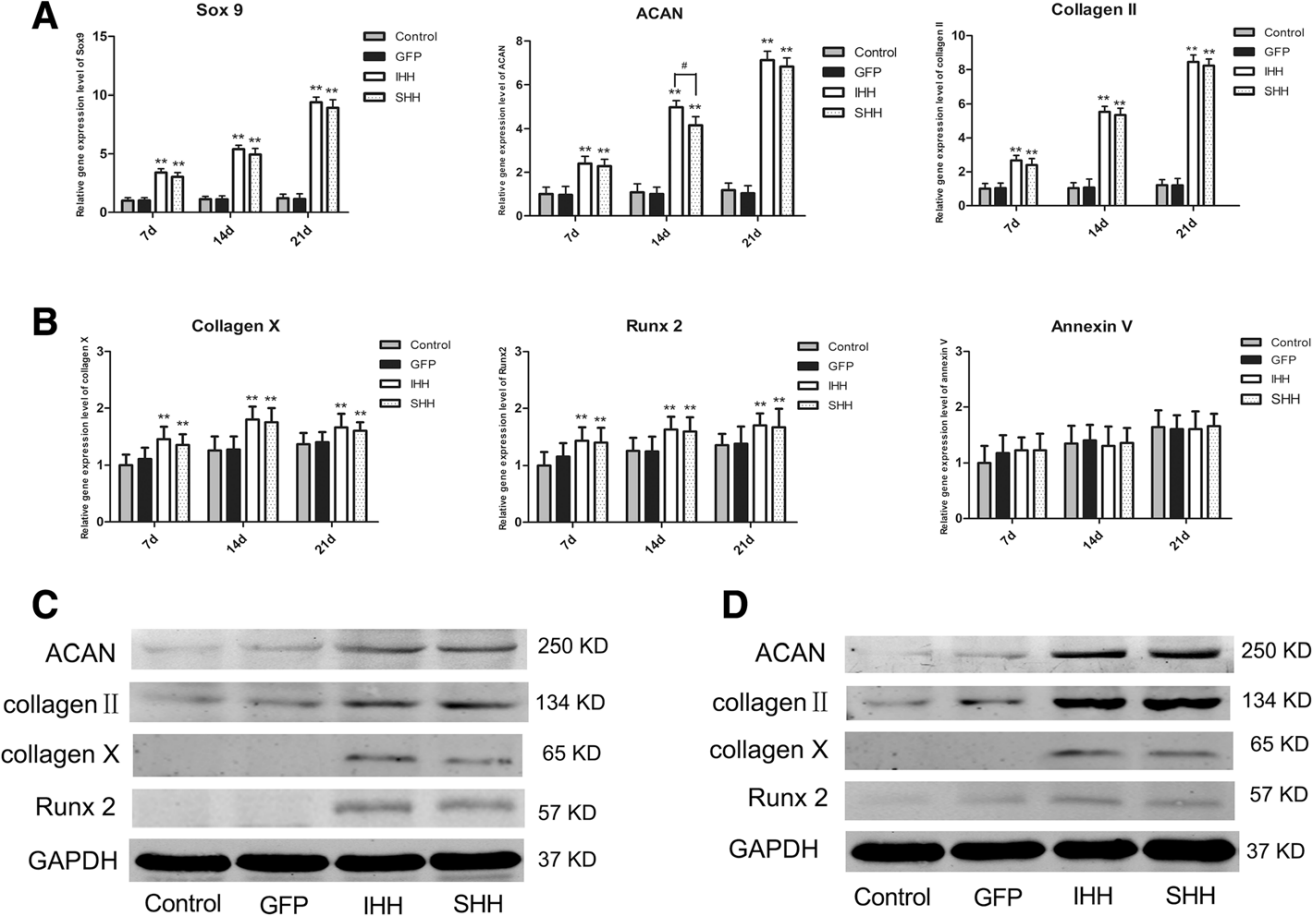
Expression levels of related genes during differentiation induction in the RCCS environment. (**a**) qPCR analysis of Sox9, ACAN and collagen II on days 7, 14 and 21 during induction. (**b**) qRT-PCR analysis of collagen X, Runx2, and annexin V on days 7, 14 and 21 during induction. The results were normalized to B2M mRNA expression. Values are means ± SD (*n* = 3). (**c**) Expression of ACAN, collagen II, collagen X and Runx2 was detected by western blotting on day 10 during differentiation induction. (**d**) Expression of ACAN, collagen II, collagen X and Runx2 was detected by western blotting on day 21 during differentiation induction. Significant differences from the control group (non-transfection cells) are indicated by *p < 0.05 or ** *p* < 0.01; differences between IHH and SHH transfection groups are indicated by ^#^ *p* < 0.05 or ^##^ *p* < 0.01

In the RCCS culture group, transfection of BMSCs with IHH and SHH induced a significant chondrogenic response over time compared to the GFP and non-transfection groups, which were not chondrogenic. The mRNA levels of the cartilage synthesis-related markers Sox9, collagen II, and ACAN in the IHH and SHH transfection groups were significantly increased during chondrogenic differentiation (*P* < 0.01) (Fig. 4a). However, no significant differences were observed between the IHH and SHH transfection groups (*P* > 0.05). Meanwhile, expression of collagen X, Runx2, and annexin V gradually increased at the early and middle stages, and plateaued at later stages (Fig. 4b). No significant differences were detected in the expression of cartilage hypertrophy-related genes in the IHH and SHH transfection groups (*P* > 0.05).

### Cartilage synthesis- and cartilage hypertrophy-related protein expression during the induction of chondrogenic differentiation

The cartilage synthesis-related proteins collagen II and ACAN and the cartilage hypertrophy-related proteins collagen X and Runx2 were detected by western blotting on days 10 and 21 after induction. In the 2D culture group, the protein levels of collagen II and ACAN in the IHH and SHH transfection groups were slightly higher than those in the GFP and non-transfection groups on day 10 (*P* < 0.01) (Fig. 3c), while both were lower on day 21 following induction of differentiation in the IHH and SHH transfection groups (Fig. 3d). On the other hand, the protein levels of collagen X and Runx2 in the IHH and SHH transfection groups were gradually increased on days 10 and 21 compared with the control groups (Fig. 3c and d).

In the RCCS group, the levels of collagen II and ACAN in the IHH and SHH transfection groups were significantly higher than those in the 2D culture groups. The levels of collagen II and ACAN in the IHH and SHH transfection groups were greatly upregulated on days 10 and 21, respectively, following induction of differentiation (*P* < 0.01; Fig. 4c and d), but no significant difference was found between the IHH and SHH transfection groups (*P* > 0.05). In addition, the expression levels of collagen X and Runx2 in the IHH and SHH transfection groups cultured in the RCCS environment were lower than those in the 2D culture environment (Fig. 4c and d).

**Fig. 5:**
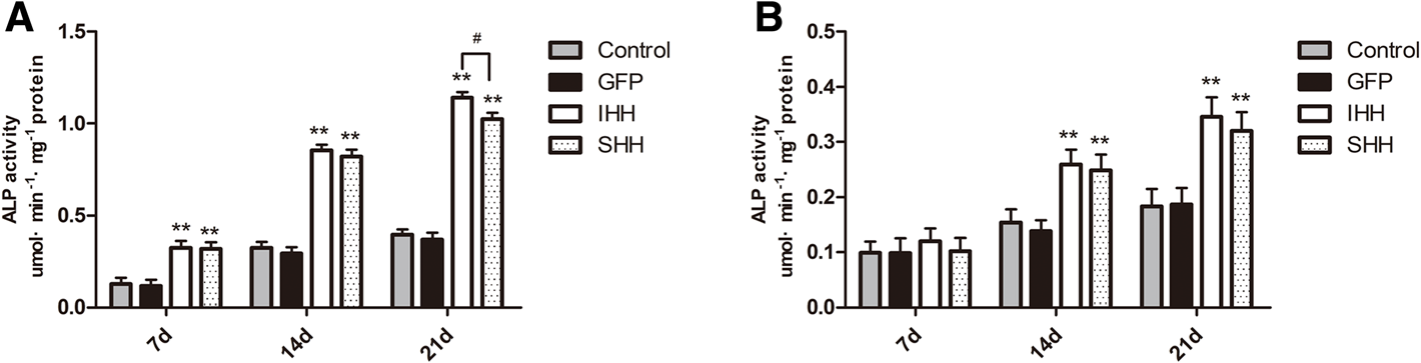
ALP activity. (**a**) ALP activity levels from each group in the 2D environment. (**b**) ALP activity levels from each group in the RCCS environment. Values are means ± SD (*n* = 3). Significant differences from the control group (non-transfection cells) are indicated by *p < 0.05 or **p < 0.01; differences between IHH and SHH transfection groups are indicated by ^#^*p* < 0.05 or ^##^*p* < 0.01

Chondrogenic hypertrophy was also quantified using ALP activity, which was significantly elevated on days 7, 14, and 21 of culture in the IHH and SHH transfection groups compared to the GFP and non-transfection groups in the traditional 2D environment (*P* < 0.01; Fig. 5a). In contrast, ALP activity was lower in the RCCS environment (Fig. 5b).

### Histological analysis and annexin V-Cy3 staining

**Fig. 6:**
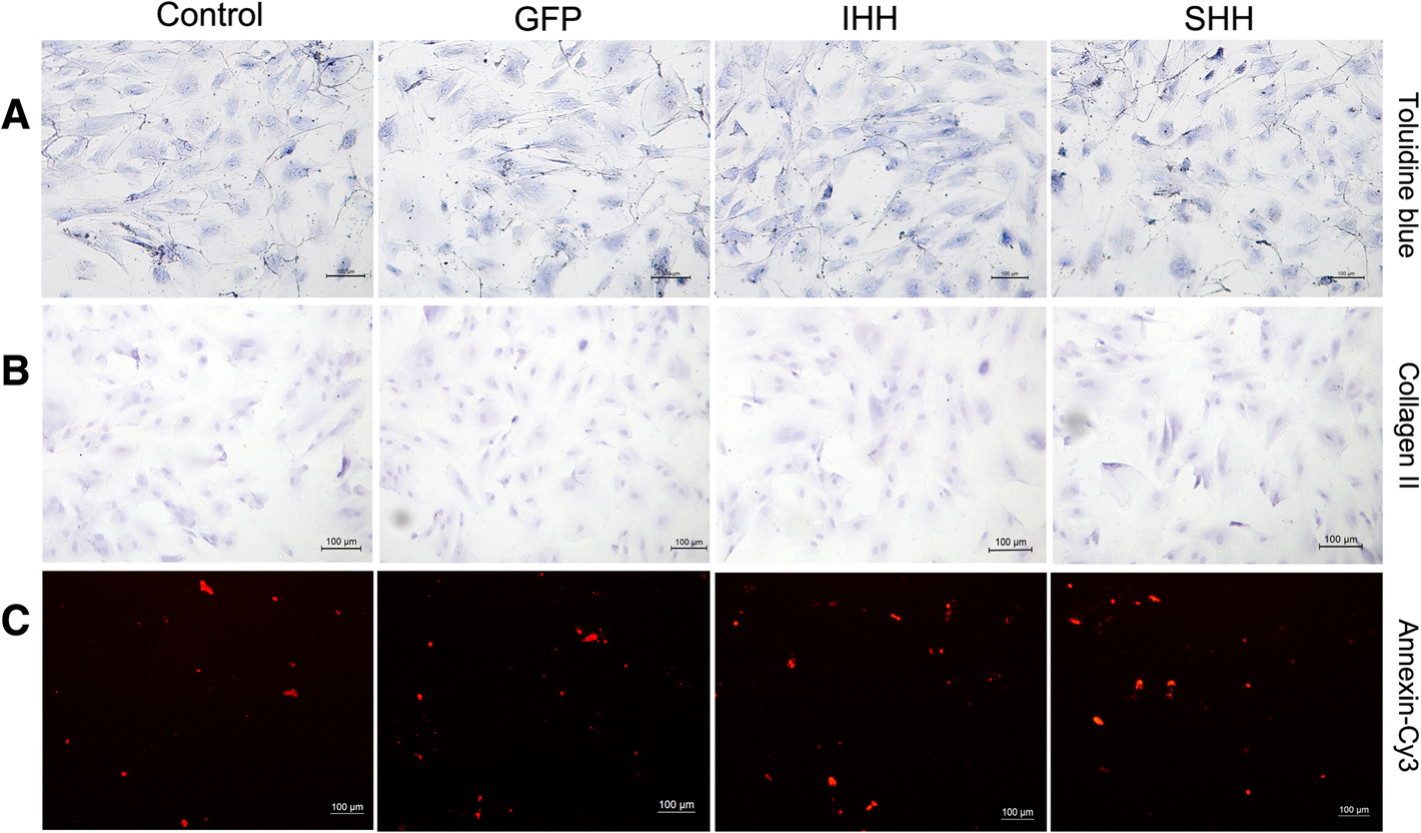
Staining results for the process of differentiation induction in the 2D environment. (**a**) Toluidine blue staining; (**b**) Collagen II immunohistochemical staining; (**c**) Annexin V-Cy3 immunofluorescence staining. (Bar = 100 um)

At day 21 following induction of differentiation, toluidine blue and collagen II immunohistochemical staining were used to test chondrogenesis, and annexin V-Cy3 immunofluorescence staining was used to test for apoptotic chondrocytes. In the 2D culture group, all groups displayed a light blue color and poor collagen II immunohistochemical staining (Fig. 6a and b). Meanwhile, all groups displayed red fluorescence following annexin V-Cy3 staining (Fig. 6c).

**Fig. 7:**
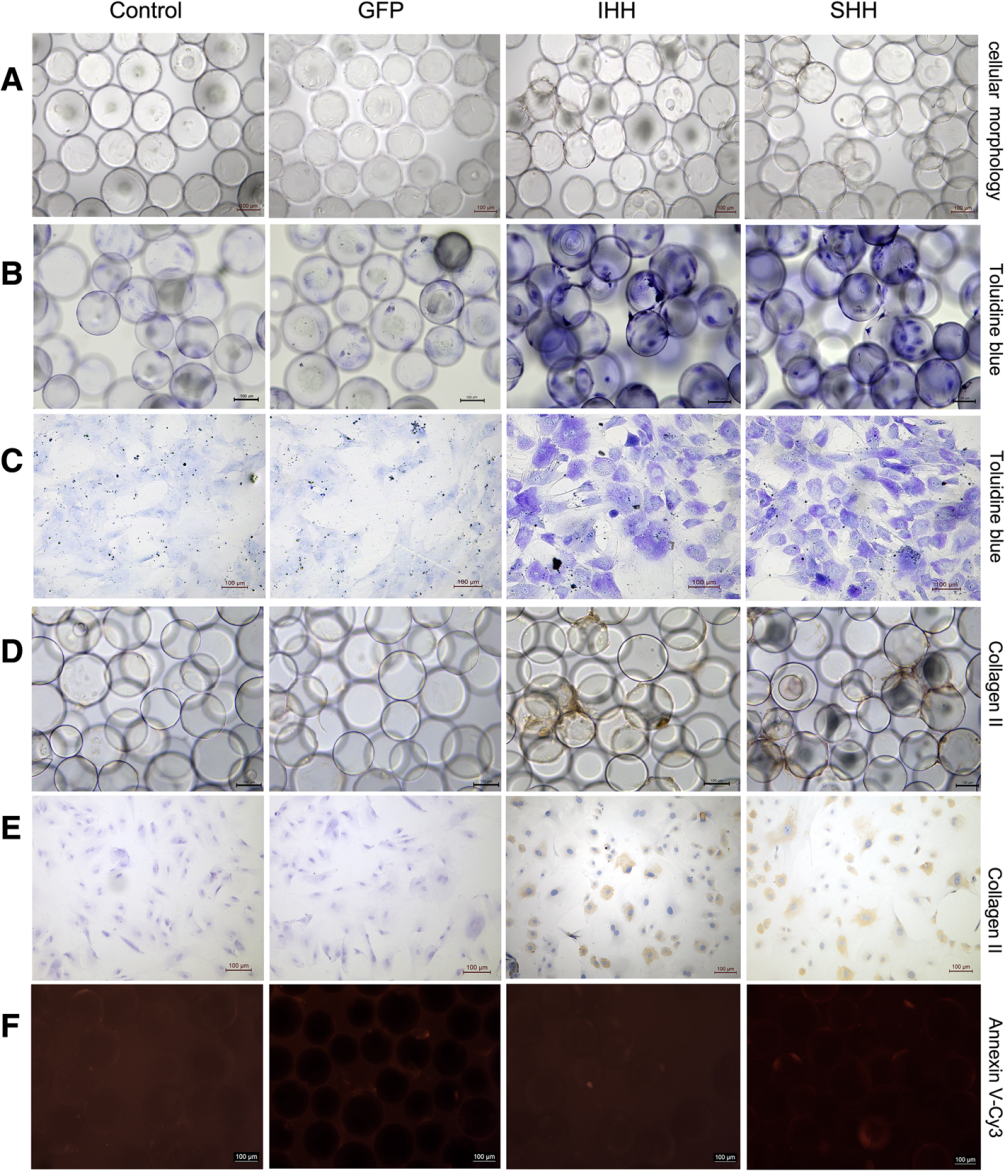
Staining results for the process of differentiation induction in the RCCS environment. (**a**) Cellular morphology. (**b**) Toluidine blue staining on cell-microcarrier complexes. (**c**) Toluidine blue staining on cells after inoculation onto slides. (**d**) Collagen II immunohistochemical staining on cell-microcarrier complexes. (**e**) Collagen II immune-histochemical staining on cells after inoculation onto slides. (**f**) Annexin V-Cy3 immunofluorescence staining. (Bar = 100 um)

Using toluidine blue staining, a stronger blue color was observed in the IHH and SHH transfection groups under microgravity conditions (Fig. 7b and c). At day 21 after differentiation, the majority of the microcarrier surface was covered with cells and abundant ECM secretion, indistinguishable single cell morphology, and large cell connection bodies between microcarriers were evident, whilst microcarriers displayed cluster growth (Fig. 7a). Staining in the IHH and SHH transfection groups was darker compared with the GFP and non-transfection groups. Furthermore, positive collagen II immunohistochemical staining was also observed in the IHH and SHH transfection groups (Fig. 7d and e). Due to widespread cellular connections between the microcarriers, there were almost no dead cells in the medium. Red fluorescence following annexin V-Cy3 staining was lower in all groups (Fig. 7f), and there was no significant difference among groups.

## Discussion

The role of hedgehog proteins in the regulation of chondrogenic differentiation and chondrogenesis has been previously established [10, 20, 21]. However, activation of hedgehog signaling also promotes chondrocyte hypertrophy and ossification during chondrogenesis, while hypertrophic differentiation and pathologic responses can affect the formation of stable hyaline cartilage [11, 12, 22, 23]. In this study, we demonstrated that chondrogenic hypertrophy and aging were markedly alleviated when chondrogenesis was induced in a microgravity culture environment. It is noteworthy that exogenous SHH and IHH had the same effect on chondrogenic differentiation as BMSCs in the RCCS environment.

Hedgehog signaling occurs through interaction of the hedgehog ligand with its receptors Ptc and Smo, which are transmembrane receptors; in the absence of hedgehog, Ptc inhibits Smo, and through suppression of Gli zinc finger transcription factors, represses downstream gene expression. When hedgehog is present, it binds to Ptc and releases Smo, which activates the hedgehog signaling pathway and allows Gli transcription factors to enter the nucleus and activate transcription of target genes [24]. In this study, the expression levels of Ptc, Smo, and Gli1 in the IHH and SHH transfection groups were significantly stimulated over time compared with the GFP and non-transfection groups. These results suggested that SHH and IHH promote chondrogenic differentiation through the same pathway [21, 25].

Chemical and mechanical stimulation is structurally and functionally required to reconstruct cartilage tissue [26]. Delivery of IHH or SHH via adenoviral vectors significantly promoted chondrogenesis of BMSCs in the RCCS environment, as shown qualitatively by toluidine blue and collagen II staining. This corresponds to findings in the same model, where adenoviral delivery of transforming growth factor (TGF)-β1 was used [27]. In contrast, the expression levels of cartilage-related markers including Sox9, ACAN, and collagen II in the IHH and SHH transfection groups were slightly higher than those in the control groups at days 7 and 14; however, the levels were significantly decreased on day 21 in the 2D culture environments. Previous studies have shown that cells may present a phenomenon similar to “dedifferentiation” in the 2D culture environment, in which they gradually lose many of the physiological characteristics of the original tissue [14, 28]. These results suggest that mechanical stimuli and hedgehog signaling act synergistically in the chondrogenic process.

Previous studies have indicated that RCCS culture of MSCs promotes proliferation and differentiation [26, 29]. In the RCCS environment, cells attach to the microcarriers cultured in a three-dimensional scaffold material and can freely rotate with rotation of the base [16]. The microcarrier composition determines its biocompatibility, and is also a crucial factor affecting cellular behavior [30]. Thus, cells and nutrients are prone to homogeneous distribution during the dynamic process of cultivation, which can significantly improve the exchange of nutrients and metabolites throughout the microenvironment, maintaining the homogeneity and stability of the culture environment and effectively promoting cell proliferation and differentiation. When BMSCs and microcarriers were co-cultured in the RCCS, we found that most cells adhered to the microcarriers. Finally, the majority of the microcarrier surface was covered with cells, with abundant ECM secretion, and large cell connection bodies between microcarriers, while microcarriers displayed cluster growth. Our findings are consistent with the observations of Kang [16], who used microcarrier technology to rapidly amplify human adipose-derived stem cell proliferation and successfully achieved chondrogenic differentiation in vitro*.*

In our previous study, we discovered the effect of chondrogenic differentiation of BMSCs transfected by IHH in the RCCS system [14]. In the current study, the RCCS also significantly enhanced the chondrogenic effect of IHH and SHH on BMSCs. We also found that SHH and IHH have the same effect on chondrogenic differentiation of BMSCs. The expression levels of Sox9, ACAN, and collagen II exhibited no significant difference between the IHH and SHH transfection groups. These results may be due to the fact that both IHH and SHH promote chondrogenesis by stimulating the Hedgehog pathway [8, 12]. In this study, we found that expression levels of Ptc, Smo, and Gli1 were significantly increased in the IHH and SHH transfection groups, while levels of Ptc, Smo, and Gli1 exhibited no significant differences between the IHH and SHH transfection groups.

The RCCS also provides a simulated microgravity environment to effectively attenuate hypertrophic differentiation and aging during chondrogenic differentiation [14]. Chondrocyte hypertrophy is part of a normal differentiation process in which cells undergo apoptosis [31], and specifically during chondrogenic differentiation in vitro. A study by Steinert et al. also showed that IHH gene transfer via adenoviral vectors alone and in combination with TGF-β1 or bone morphogenetic protein (BMP)-2 efficiently induces chondrogenesis, but also promotes hypertrophy [17]. In this study, we found that the IHH and SHH proteins facilitate the progression of chondrogenesis at early stages, and promote hypertrophic differentiation and aging in the traditional 2D culture environment. The expression of the chondrocyte hypertrophy and aging-related genes Runx2, collagen X, and annexin V in the IHH and SHH transfection groups was significantly increased when cultured in the traditional 2D environment at day 21 following chondrogenic induction. Previous investigations have revealed that forced overexpression of IHH or SHH might have resulted in an imbalance within the finely tuned regulatory system with parathyroid hormone-related protein (PTHrP) that may have influenced the missing modulation of hypertrophy [6, 17, 22]. Therefore, suppressing chondrocyte hypertrophy induced by IHH and SHH during chondrogenic differentiation is important in the generation of stable hyaline cartilage.

Under conditions of microgravity, culture materials can establish a suspension track similar to a homogeneous fluid on a horizontal axis, and the gravity, buoyancy, and shear force can achieve a balance, which constitutes a microgravity environment conducive to cell aggregation. The correct hydrostatic pressure and shear stress also aid in maintaining the phenotype and function of cartilage cells [16, 32]. Our data demonstrated that Runx2, collagen X, and annexin V gene expression levels were lower in the IHH and SHH transfection groups under microgravity conditions compared with the 2D culture group, indicating that microgravity markedly attenuated hypertrophic differentiation and aging in the RCCS environment. Based on these findings, we believe that the continued mechanical stress due to the medium flow and “low shear modeled microgravity” would be beneficial to inhibit chondrogenic hypertrophy and aging induced by IHH and SHH during chondrogenesis. On the other hand, in this dynamic environment, the enhanced cell activity coupled with the inductive factors leads to increased production and secretion of proteins that form the ECM and promote the differentiation and maturation of cells [16].

The results of our investigation also have several limitations. First, our experiments are limited to in vitro scenarios, and further studies should be performed in vivo. Moreover, several other signaling systems have been found to interact with IHH or SHH during chondrogenic differentiation [12, 33, 34]. Previous studies have suggested that IHH is also important for maintaining endogenous TGF-β and BMP signaling, and collectively, these three signaling pathways may work together to drive optimal chondrogenesis [35]. However, TGF-β also acts as a signal relay between IHH and PTHrP in the regulation of hypertrophic cartilage differentiation [36]. Further studies should be undertaken to investigate these signaling cascades in the RCCS environment.

## Conclusions

In summary, the present study showed that microgravity significantly promoted exogenous SHH and IHH in chondrogenic differentiation of BMSCs and effectively attenuated chondrogenic hypertrophy and aging induced by IHH and SHH during chondrogenic differentiation. Furthermore, exogenous SHH and IHH had the same effect on chondrogenic differentiation of BMSCs in the RCCS environment.

## Additional file

Additional file 1: **Figure S1.** BMSC identification. (PDF 214 kb)

## Abbreviations

ACAN: Aggrecan
BMSCs: Bone marrow-derived mesenchymal stem cells
ECM: Extracellular matrix
GAPDH: Glyceraldehyde 3-phosphate dehydrogenase
GFP: Green fluorescence protein
IHH: Indian hedgehog
PTHrP: Parathyroid hormone-related protein
qPCR: Reverse transcription real-time PCR
RCCS: Rotary cell culture system
SHH: Sonic hedgehog
