# Supplementary material for: Chondrogenic differentiation of bone marrow-derived mesenchymal stem cells following transfection with Indian hedgehog and sonic hedgehog using a rotary cell culture system
Source: Cell Mol Biol Lett. 2019 Feb 26;24:16. doi: 10.1186/s11658-019-0144-2 (PMC6390628; doi:10.1186/s11658-019-0144-2)
Supplement: Supplementary file 1 — Figure S1. BMSC identification. (PDF 214 kb) [file 11658_2019_144_MOESM1_ESM.pdf]

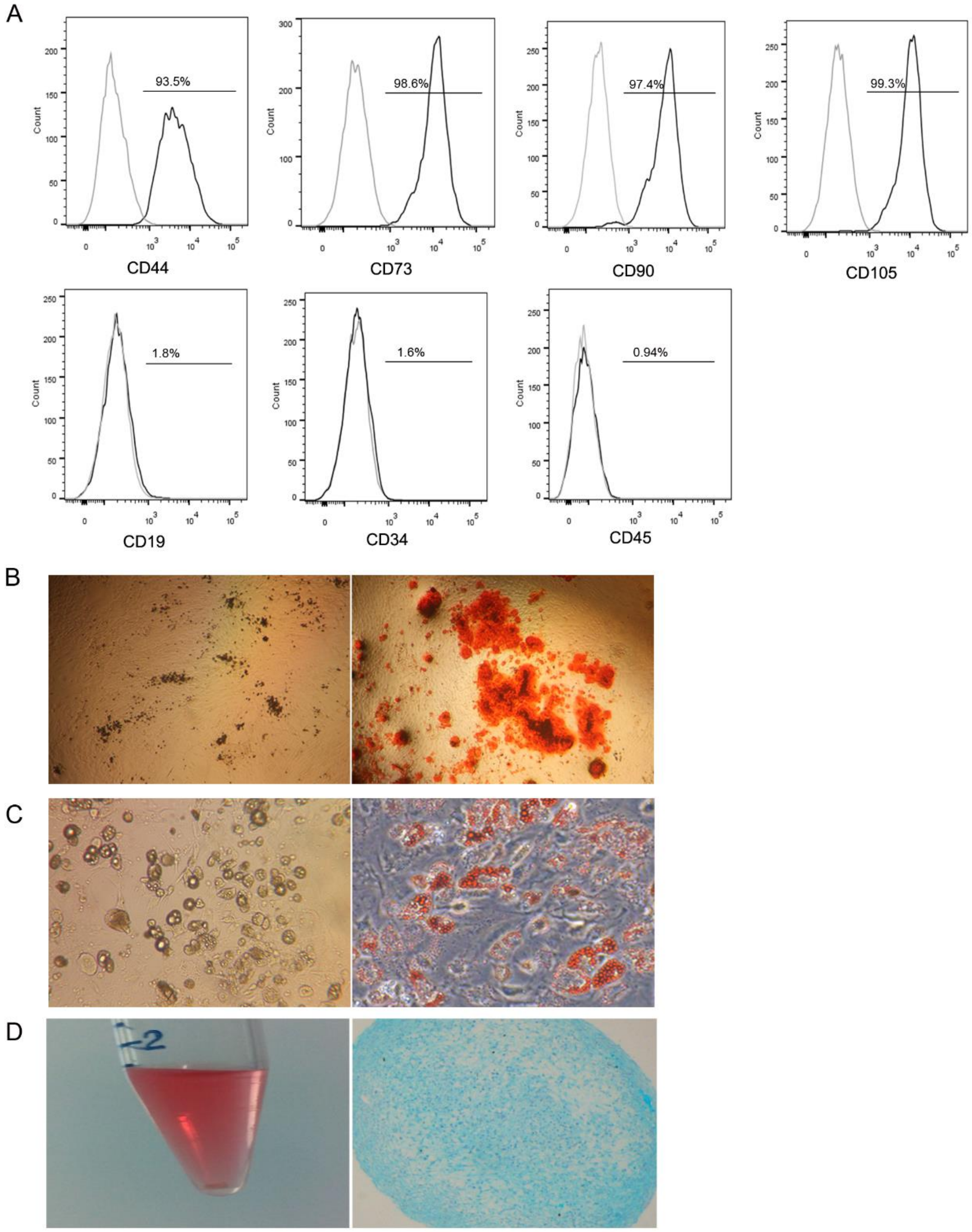

Fig. S1 BMSC identification. **(a)** Graphs showing expression of mesenchymal stem cell markers. High percentage of cells expressed CD44, whereas only few cells expressed CD45; **(b)** Osteogenic differentiation; **(c)** Adipogenic differentiation. **(d)** Chondrogenic differentiation;
